# Antibacterial, Antiparasitic, and Cytotoxic Activities of Chemical Characterized Essential Oil of *Chrysopogon zizanioides* Roots

**DOI:** 10.3390/ph15080967

**Published:** 2022-08-05

**Authors:** Thaís A. S. Oliveira, Tatiana M. Vieira, Viviane R. Esperandim, Carlos H. G. Martins, Lizandra G. Magalhães, Mayker L. D. Miranda, Antônio E. M. Crotti

**Affiliations:** 1 Department of Chemistry, Faculty of Phylosophy, Sciences, and Letters of Ribeirão Preto, University of São Paulo, Av. Bandeirantes, 3900, Ribeirão Preto 14049-900, SP, Brazil; 2 University of Franca, Av. Dr. Armando Salles Oliveira, 201—Parque Universitário, Franca 14404-600, SP, Brazil; 3 Institute of Biomedical Sciences, Federal University of Uberlândia, Rua Ceará, 1720, Umuarama, Uberlândia 38402-018, MG, Brazil; 4 Federal Institute of Education, Science, and Technology of Triângulo Mineiro, Campus Uberlândia, Centro, Rua Blanche Galassi, 150, Morada da Colina, Uberlândia 38411-104, MG, Brazil

**Keywords:** Chrysopogon zizanioides, β-eudesmol, khusimol, Leishmania amazonensis, periodontopathogens, Trypanosoma cruzi

## Abstract

This study aimed to investigate the chemical composition as well as the antibacterial, antiparasitic, and cytotoxic potentialities of the Brazilian *Chrysopogon zizanioides* root essential oil (CZ-EO) In addition, CZ-EO cytotoxicity to LLCMK_2_ adherent epithelial cells was assessed. The major compounds identified in CZ-EO were khusimol (30.0 ± 0.3%), β-eudesmol (10.8 ± 0.3%), α-muurolene (6.0 ± 0.1%), and patchouli alcohol (5.6 ± 0.2%). CZ-EO displayed optimal antibacterial activity against *Prevotella nigrescens*, *Fusobacterium nucleatum*, *Prevotella melaninogenica*, and *Aggregatibacter actinomycetemcomitans*, with Minimum Inhibitory Concentration (MIC) values between 22 and 62.5 µg/mL and Minimum Bactericidal Concentration (MBC) values between 22 and 400 µg/mL. CZ-EO was highly active against the *L. amazonensis* promastigote and amastigote forms (IC_50_ = 7.20 and 16.21 µg/mL, respectively) and the *T. cruzi* trypomastigote form (IC_50_ = 11.2 µg/mL). Moreover, CZ-EO showed moderate cytotoxicity to LLCMK_2_ cells, with CC_50_ = 565.4 µg/mL. These results revealed an interesting in vitro selectivity of CZ-EO toward the *L. amazonensis* promastigote and amastigote forms (Selectivity Index, SI = 78.5 and 34.8, respectively) and the *T. cruzi* trypomastigote form (SI = 50.5) compared to LLCMK_2_ cells. These results showed the promising potential of CZ-EO for developing new antimicrobial, antileishmanial, and antitrypanosomal drugs.

## 1. Introduction

Gingivitis and periodontitis, both periodontal diseases characterized by a chronic infection associated with bacteria living in the oral cavity, cause irreversible destruction of tissues supporting the teeth [1]. Gingivitis, an inflammatory process occurring in the soft tissues surrounding the teeth (gum), is an immune response to plaque that these bacteria form on the tooth. Because periodontitis results in tooth loss, the disease is considered more aggressive than gingivitis [2]. In the United States, the prevalence of periodontitis was estimated at 45.9% between 2009 and 2012; 8.9% of the cases corresponded to severe periodontitis [3]. Among over 300 species of periodontopathogenic bacteria, *Aggregatibacter actinomycetemcomitans*, *Prevotella melaninogenica*, *Prevotella nigrescens*, and *Fusobacterium nucleatum* can be highlighted [4].

Trypanosomiasis, or Chagas disease, is a serious public health problem that affects between 6 and 7 million people. This Neglected Tropical Disease (NTD) predominates in Latin American and Caribbean countries, where prevention and treatment have progressed very slowly. However, globalization and migration have caused the number of cases to increase worldwide, including in developing countries [5]. Chagas’s disease is caused by *Trypanosoma cruzi*. This protozoan is transmitted to humans when feces of insects contaminated with this microorganism penetrate a skin wound or mucosa. Infection can also occur by vertical, blood, or oral transmission. In the latter case, transmission is possible when the feces of infected vectors or macerated insects contaminate fresh fruit juices or other foods [5]. Since the 1970s, nifurtimox (Lampit) and benznidazole (Rochagan) have been the only drugs available for treating Chagas disease. These drugs have side effects that include anorexia, nausea, vomiting, headache, central nervous system depression or manic symptoms, convulsions, vertigo, paresthesia, peripheral polyneuropathies, and dermatitis, which leads to 10–30% of the patients to discontinue treatment [6].

Leishmaniasis is another NTD that impacts global public health, with an estimated 30,000 annual deaths. This complex disease is caused by different species of protozoa belonging to the genus *Leishmania*, leading to tegumentary (TL) or visceral (VL) manifestations depending mainly on the infecting *Leishmania* species and the mammalian host’s immunological and nutritional status. Tegumentary leishmaniasis (TL) can be subclinical or cause self-healing skin lesions that can culminate in disfiguring scars, accompanied by extensive tissue destruction in nasopharyngeal mucosal tissues. [7]. In the Americas, *Leishmania amazonensis* is one of the main species underlying TL, mainly cutaneous leishmaniasis (CL) and diffuse cutaneous leishmaniasis (DCL). TL evolves slowly, covering large skin areas and making an initial diagnosis and treatment difficult [8]. The standard treatment against leishmaniasis is based on pentavalent antimonials and second-line drugs, such as free or liposomal amphotericin B, pentamidine, miltefosine, and paramomycin. However, these drugs have serious disadvantages that limit their effectiveness, including toxicity, high cost, and emergence of parasitic resistance [7].

Essential oils (EOs) are mixtures of volatile secondary metabolites that can be obtained from different parts (e.g., leaves, flowers, seeds, bark, fruits, and roots) of medicinal and aromatic plants. EOs display diverse biological activities, like antimicrobial [9], antileishmanial [10,11], and trypanocidal [12] action. Some of these activities have attracted researchers’ attention.

*Chrysopogon zizanioides* (L.) Roberty (Poaceae), a synonym of *Vetiveria zizanioides* (L.) Nash, is native to the tropical regions of northern India, but it is distributed worldwide wherever the environmental conditions are appropriate for its development [13]. The culms are erect, and it resembles lemongrass. *C. zizanoides* roots are strong, spongy, and aromatic, and their distribution is massive and compact. They are useful for treating hyperdipsia, burning sensations, skin diseases, nausea, vomiting, dyspepsia, flatulence, bilious fever, gout, lumbago, sprains, halitosis, cephalalgia, amentia, amenorrhea, helminthiasis, and general debility [14]. The EO of *C. zizanioides* is commonly known as “tranquility oil” or “vetiver oil” and has significant commercial importance due to its unique fragrance, which is not provided by any other synthetic compound. Because of its fragrance, the EO of *C. zizanioides* is commonly used in foods. This species is also used for treating ulcers, fever, headache, inflammation, and gastritis [15].

As part of our ongoing research into the antimicrobial [16,17,18] and antiparasitic [19,20] activities of EOs, the current study describes the in vitro leishmanicidal and trypanocidal activities of the EO from *C. zizanoides* roots collected in Southeastern Brazil (CZ-EO), as well as its antimicrobial action against periodontopathogenic bacteria. We also investigate the chemical composition and the cytotoxicity of this EO to LLCMK_2_ adherent epithelial cells.

## 2. Results

### 2.1. CZ-EO Chemical Composition

Table 1 displays the chemical composition of the essential oil from *C. zizanioides* roots (CZ-EO), as determined by gas chromatography with flame ionization detection (GC-FID) and gas chromatography-mass spectrometry (GC–MS). A total of 22 compounds were identified, most of which (70.4 ± 0.1 %) are oxygenated sesquiterpenes. The major compounds in CZ-EO are khusimol (30.0 ± 0.3%), β-eudesmol (10.8 ± 0.3%), α-muurolene (6.0 ± 0.1%), and patchouli alcohol (5.6 ± 0.2%). We did not detect monoterpenes or phenylpropanoids in CZ-EO.

### 2.2. CZ-EO Antibacterial Activitiy against Periodontopathogenic Bacteria

The Minimum Inhibitory Concentration (MIC) values obtained for CZ-EO ranged from 22.0 ± 6.25 to 250.0 ± 100.0 µg/mL against all the studied bacteria (Table 2). This EO showed very strong activity against seven out of nine investigated bacteria, namely *P. intermedia* ATCC 49046 (MIC = 22.0 ± 6.25 µg/mL), *A. actinomycetemcomitans* ATCC 43717 (MIC = 22.0 ± 6.25 µg/mL), *P. melaninogenica* ATCC 700524 (MIC = 50.0 ± 0.0 µg/mL), *F.* *nucleatum* (MIC = 50.0 ± 0.0 µg/mL), *P. gingivalis* ATCC 33277 (MIC = 62.5 ± 25.0 µg/mL), and *P. nigrescens* ATCC 33563 (MIC = 62.5 ± 25.0 µg/mL). CZ-EO also exhibited strong activity against *P. gingivalis* Clinical Isolate (MIC = 100.0 ± 0.0 µg/mL), *P. intermedia* Clinical Isolate (MIC = 150.0 ± 58.0 µg/mL), and *F. nucleatum* Clinical Isolate (MIC = 250.0 ± 100.0 µg/mL) (Table 2).

Regarding the Minimum Bactericidal Concentration (MBC), the MBC values obtained for CZ-EO against *A. actinomycetemcomitans* (22.0 ± 6.25 µg/mL), *F. nucleatum* (50.0 ± 0.0 µg/mL), *P. melaninogenica* (50.0 ± 0.0 µg/mL), and *P. nigrescens* (62.5 ± 25.0 µg/mL) were the same as the respective MIC values (Table 2). In the case of *P. gingivalis* (ATCC 33277 and clinical isolate), *P. intermedia* (ATCC 49046 and clinical isolate), and *F. nucleatum* (clinical isolate), the MBC values were higher compared to the MIC values.

### 2.3. CZ-EO Antileishmanial, Trypanocidal, and Cytotoxic Activities

Table 3 shows the results obtained during evaluation of the leishmanicidal activity of CZ-EO against the *L. amazonensis* promastigote and amastigote forms. The half-maximum inhibitory concentration (IC_50_) values of 7.20 and 16.21 μg/mL against the *L. amazonensis* promastigote and amastigote forms, respectively, are promising compared to amphotericin B (IC_50_ = 0.25 μg/mL).

As for the trypanocidal activity, the IC_50_ value obtained for CZ-EO against the *T. cruzi* trypomastigote form was 11.2 μg/mL. (Table 4). Benznidazole, used as a positive control, displayed IC_50_ = 9.9 µg/mL. CZ-EO exhibited moderate toxicity to the LLCMK_2_ cells (CC_50_ = 565.4 μg/mL) and Selectivity Index (SI = CC_50_/IC_50_) values of 78.5, 34.8, and 50.4 against the *L. amazonensis* promastigote and amastigote forms and *T. cruzi* trypomastigote forms, respectively (Table 4).

## 3. Discussion

Sesquiterpenes have been reported as the major compounds in *C. zizanioides* EOs; oxygenated sesquiterpenes, especially alcohols (18–49%) (e.g., kushimol and vetiselinenol) and ketones (9–14%) (e.g., the α- and β-vetivones, kushimone, and nootkatone), predominate in these EOs [13]. Here, we have only identified sesquiterpenes in CZ-EO, with khusimol (30.0 ± 0.3%) and β-eudesmol (10.8 ± 0.3%) being the major compounds (Table 1). Of the identified sesquiterpenes, 70.4 ± 0.1% are oxygenated, and 24.6 ± 0.5% are hydrocarbons. This finding corroborates the finding of Champagnat and co-workers, who investigated the chemical composition of the commercial EOs from *C. zizanoides* collected in nine different countries [13,22,23]. After using principal component analysis (PCA), these authors concluded that the chemical composition of these EOs is relatively homogeneous, with the major compounds being khusimol, β-vetienene, and β-vestipirene [23]. More recently, Lunz & Stappen reviewed the literature on the chemical composition of the EO from *C. zizanioides* roots. Although they reported that about 300 compounds have been detected in this EO, khusimol, β-vetispirene, vetiselinenol, and α-vetivone have always been the major compounds in the EO from *C. zizanioides* roots throughout the world [15].

The antimicrobial activity of the EO from *C. zizanioides* roots has been investigated against a broad range of bacteria including (*Staphylococcus aureus* [24], *Staphylococcus saprophyticus* [25], *Staphylococcus pyogenes* [25], *Enterococcus faecalis* [26], *Escherichia coli* [26], *Mycobacterium tuberculosis* [26]) and fungi (e.g., *Aspergillus fumigatus*, *Microsporum canis*, *Trichophyton rubrum*, and *Candida albicans*) [24]. However, the antibacterial activity of this EO against periodontopathogenic bacteria has not been reported.

According to the literature, EOs with Minimum Inhibitory Concentration (MIC) values less than or equal to 100 µg/mL, between 101 and 500 µg/mL, between 501 and 1500 µg/mL, and between 1500 and 2000 µg/mL denote very strong, strong, moderate, and weak activity, respectively [9,27,28]. On the basis of these criteria, CZ-EO displays very strong activity against most of the periodontopathogenic evaluated herein (Table 2). In addition, CZ-EO showed a bacteriostatic effect against five of the evaluated bacteria (for which the MBC values were higher than the MIC values) and bactericidal for four of the periodontal bacteria tested (for which the values of the MIC and MBC concentration were the same) [29]. This activity can be due to the presence of β-eudesmol, one of the major compounds in CZ-EO. This compound can inhibit glucosyltransferase and reduce dental caries, as recently reported [30]. This result is noteworthy because the risk of pathogenic microorganisms developing resistance to EOs is very low, as EOs contain a blend of antimicrobial compounds with different modes of action [31,32].

Regarding the leishmanicidal and trypanocidal activities, the literature describes that EOs with IC_50_ lower than 10 μg/mL, between 10 and 50 μg/mL, between 50 and 100 μg/mL, and higher than 100 μg/mL are very active, active, moderately active, and inactive [33,34,35]. On the basis of these criteria, CZ-EO is highly active against the *L. amazonensis* promastigote forms (IC_50_ = 7.20 ± 1.14 µg/mL) and active against the *L. amazonensis* amastigote forms (IC_50_ = 16.21 ± 1.02 µg/mL). In addition, CZ-EO is active against the *T. cruzi* trypomastigote form (IC_50_ = 11.2 µg/mL), with its IC_50_ resembling the IC_50_ obtained for benznidazole (IC_50_ = 9.9 µg/mL), used as a positive control. Concerning cytotoxicity, values of 50% cytotoxic concentration (CC_50_) lower than 10 μg/mL, between 10 and 100 μg/mL, between 100 and 1000 μg/mL, and higher than 1000 μg/mL are typical of highly toxic, toxic, moderately toxic, and non-toxic EOs [36]. Thus, CZ-EO displays moderate cytotoxicity to LCCK2 cells (CC_50_ = 565.4 ± 1.0 μg/mL) (Table 4).

Recently, Monzote and co-workers investigated the in vitro leishmanicidal activity of a commercially available sample of the EO from *C. zizanoides* against the *L. amazonensis* promastigote form. Although the authors did not specify from which part of the plant the EO was obtained, they reported an IC_50_ of 19.0 ± 3.3 µg/mL and a CC_50_ = 31.7 ± 2.8 µg/mL, which denotes unspecific activity (SI = 2) [37]. Here, we obtained IC_50_, CC_50_, and SI values of 7.20 ± 1.14 µg/mL, 565.4 ± 1.0 µg/mL, and 78.5 for CZ-EO, respectively. These results indicate that CZ-EO is more active compared to the literature—not only does it display a considerably lower IC_50_ value, but it also has an almost 40 times higher selectivity index (SI). Even though these differences can be due to the distinct susceptibilities of the *L. amazonensis* strains and the cell lineages used in the antileishmanial and cytotoxic assays conducted herein, they might also be due to differences in the chemical composition of CZ-EO and the commercial EO investigated by Monzote and co-workers [37]. However, data on the chemical composition of the EO of *C. zizanoides* were not reported by the authors [37]. On the other hand, this is the first report on the in vitro antileishmanial activity of the EO from *C. zizanioides* against the *L. amazonensis* promastigote form. The activity displayed by CZ-EO against the *L. amazonensis* amastigote form is also a noteworthy result because amastigotes are the parasite forms that persist in the infected host [38].

The biological activities of EOs can be due to the bioactivities of their components. β-eudesmol, one of the main compounds in CZ-EO, displays antitrypanosomal activity (EC_50_ = 5.45 µg/mL) against *Trypanosoma brucei brucei* GUTat 3.1 and displays low cytotoxicity to MRC-5 cells (CC_50_ > 100 µg/mL, SI > 18) [39]. Bailen and co-workers reported the antileishmanial and trypanocidal activities of β-eudesmol against *Leishmania infantum* (IC_50_ = 52.5 μg/mL) and *Trypanosoma cruzi* (IC_50_ > 100 μg/mL) [40]. The EOs from *Guatteria friesiana* and *Guatteria pogonopus* leaves, which are rich in β-eudesmol, display trypanocidal action against the *T. cruzi* bloodstream trypomastigote and epimastigote forms, with IC_50_ values of 11.9 and 28 μg/mL against epimastigotes and 10.7 μg/mL and 41.3 μg/mL against trypomastigotes, respectively [41]. On the other hand, both the antimicrobial and antiparasitic effects cannot be associated with the presence of β-eudesmol only: additive and synergistic effects between β-eudesmol and other CZ-EO components can also occur.

## 4. Materials and Methods

### 4.1. Plant Material

*Chrysopogon zizanioides* (L.) Roberty (Poaceae) roots were collected in June 2021, in the municipality of Machado, State of Minas Gerais, Southeastern Brazil (21°41′56″ S and 45°52′59″ W). The plant material was identified by the botanist Walnir G. F. Júnior. A voucher specimen (GERAES-CZ01) was deposited at Herbário de Machado of the Departamento de Biologia, Instituto Federal de Educação, Ciência e Tecnologia do Sul de Minas Gerais, Brazil.

### 4.2. CZ-EO Distillation

*C. zizanoioides* roots (1500 g) were divided into three samples (500 g each) and accommodated into three 500 mL round bottom flasks. Then, 500 mL distilled water was added, and the flask was coupled to a Cleavenger-type apparatus. After hydrodistillation for 4 h, CZ-EO was obtained as a light-yellow oil in a 0.52 ± 0.04% yield (*w/w*). Next, the EO was dried over MgSO_4_ and stored at −4 °C until GC analyses and biological assays were performed.

### 4.3. Identification of CZ-EO Compounds

CZ-EO was dissolved in ethyl ether and analyzed by gas chromatography flame- ionization detection (GC-FID) and gas chromatography-mass spectrometry (GC–MS) on the Shimadzu QP5000 Plus and GCMS2010 Plus (Shimadzu Corporation, Kyoto, Japan) systems, respectively. During GC-FID, the column temperature was programmed to rise from 60 to 240 °C at 3 °C/min and held at 240 °C for 5 min. H_2_ was used as the carrier gas at a flow rate of 1.0 mL/min. The equipment was set to operate in the injection mode; the injection volume was 0.1 µL (split ratio of 1:10); and the injector and detector temperatures were 240 and 280 °C, respectively. Relative concentrations of the CZ-EO compounds were estimated from the relative peak areas (%) of the GC-FID chromatograms, and expressed as the average of triplicate analyses and the corresponding standard deviations. GC-MS conditions and the identification were based on a previously reported methodology [17]. The volatile components in CZ-EO (Table 1) were identified on the basis of their retention indices on an Rtx-5MS (30 m × 0.25 mm; 0.250 µm) capillary column, under the same operating conditions used for the GC relative to a homologous series of n-alkanes (C_8_–C_20_). The structures were computer matched with Wiley 7, NIST 08, and FFNSC 1.2, and their fragmentation patterns were compared with literature data [21].

### 4.4. Bacterial Strains and Antimicrobial Assays

To test CZ-EO against periodontal pathogens, six standard strains from the American Type Culture Collection (ATCC, Manassas, VA, USA) (*Porphyromonas gingivalis* ATCC 33277; *Aggregatibacter actinomycetemcomitans* ATCC 43717, *Prevotella intermedia* ATCC 49046, *Prevotella nigrescens* ATCC 33563, *Fusobacterium nucleatum* ATCC 25586, and *Prevotella melaninogenica* ATCC 700524) and three clinical isolates (*P. gingivalis*, *F. nucleatum*, and *P. intermedia*) were used. The evaluated clinical isolates were obtained from clinical trials and kept in the Laboratory of Antimicrobial Testing library under cryopreservation at −20 °C. The Minimum Inhibitory Concentration (MIC) values were determined using the microdilution broth method in 96-well microplates (TPP, Trasadingen, Switzerland) [42]. To this end, samples of CZ-EO at concentrations ranging from 0.195 to 400.0 µg/mL were tested. Next, the anaerobic bacteria were incubated at 37 °C for 72 h in an anaerobic workstation (Don Whitley Scientific, Bradford, UK) with 5 or 10% H_2_, 10% CO_2_, 85 or 80% N_2_ atmosphere. After that, resazurin (Sigma-Aldrich, St. Louis, MO, USA) (30 µL) in an aqueous solution (0.02%) was added to the microplates to indicate microorganism viability [43]. Before resazurin was added and to determine the MBC, an aliquot of the inoculum (10 µL) was aseptically removed from each well and plated onto Brucella agar supplemented with 5% sheep blood, hemin (5 mg/mL, Sigma, St. Louis, MO, USA) and menadione (1 mg/mL). The plates were incubated as described previously. The Minimum Bactericidal Concentration (MBC) was determined as the lowest concentration of CZ-EO at which no bacterial growth occurred. The MIC and MBC were determined in quadruplicate, and the results were presented as a mean ± standard deviation.

### 4.5. Antileishmanial Assays

The antileishmanial activity of CZ-EO against *L. amazonensis* (MHOM/BR/PH8) promastigote forms was evaluated according to the previously described methodology [44]. The assays were performed on a 96-well microplate containing 1 × 10^6^ parasites per well. A stock solution of CZ-EO dissolved in DMSO (Sigma-Aldrich, St Louis, MO, USA) (1 mg/mL) was added to the cultures to achieve final concentrations from 3.12 to 50 µg/mL. Amphotericin B (Sigma-Aldrich, 97% purity) at concentrations of 0.19 to 0.011 µg/mL was used as the positive control. RPMI 1640 medium (Gibco-Life Technologies, Grand Island, NE, USA), and RPMI 1640 culture plus 0.1% DMSO (the highest non-toxic concentration) was used as the negative control. Cultures were incubated at 25 °C in BOD ovens (Quimis) for 24 h. The leishmanicidal activity was determined based on the growth inhibition of promastigote forms by counting the total number of alive promastigotes in the Neubauer chamber (Global Glass, Porto Alegre, Brazil) considering their flagellar motility. Results were expressed as the mean of the lysis percentage relative to the negative control [45,46]. The experiments were performed in triplicate. The maintenance of life cycle was approved by the Ethics Committee for Animal Care at the University of Franca, under protocol number 010/14.

The antileishmanial activity of CZ-EO against amastigote forms of *L.* (L.) *amazonensis* was assessed as described by Casa and co-workers, with modifications [47]. Briefly, macrophages cultivated in a supplmented RPMI 1640 medium were suspended and adjusted to a concentration of 2 × 10^5^ cells/well and seeded onto a 96-well round-coverslip culture plate. They were incubated for 96 h at 37 °C in the presence of a 5% CO_2_ atmosphere. Adherent macrophages were infected with metacyclic promastigotes (stationary growth phase, 5-day culture) at a concentration of 1 × 10^6^ cells/well (10:1 ratio) for 4 h at 37 °C. Parasites that were not internalized in the macrophages were removed, and the infected cultures were incubated with different concentrations of CZ-EO (3.12 to 50 µg/mL) and Amphotericin B (0.011 to 0.19 μg/mL) for 48 h at 37 °C and 5% CO_2_. After incubation, the coverslips were washed, fixed with methanol, and stained with Giemsa solution (Synth). The slides were analyzed in an optical microscope (Nikon New York, New York, NY, USA) by counting 200 macrophages and which determined the number of amastigote forms within each infected cell. The EC_50_ was calculated in comparison to the negative control (RPMI 1640 medium containing 0.1% DMSO).

### 4.6. Trypanocidal Assay

The in vitro trypanocidal assays were performed using *Trypanosoma cruzi* (Y strain) trypomastigote forms. This strain has been maintained at the University of Franca Vivarium through successive tests in Swiss mice by cardiac puncture on the day of the parasitemia peak (seventh day of infection). This procedure was approved by the National Council for Control of Animal Experimentation of the Ethics Committee at the University of Franca, under protocol number 010/14. The assay was performed with blood from infected albino mice by cardiac puncture at the parasitemia peak (seventh day of infection). The infected blood was diluted with a physiological solution to achieve a final blood concentration of 1 × 10^6^ trypomastigote forms/mL. Aliquots of CZ-EO diluted in DMSO (1 mg/mL) were added to the infected blood on the microtiter plate (96 wells) to achieve a final volume of 200 μL containing CZ-EO at final concentrations from 12.5 to 200 µg/mL. Benznidazole (Roche, Rio de Janeiro, RJ, Brazil) and 0.5% DMSO (the highest non-toxic concentration for this cell type) were used as the positive and negative controls, respectively. The microplate was incubated at 37 °C for 24 h. The activity was quantitatively verified by counting the trypomastigote forms as previously described in the literature [48]. The parasite lysis percentage was determined by comparison with the control group without treatment. Two experiments were performed in triplicate.

### 4.7. Cytotoxicity Assay

The LLCMK_2_ cells were counted in a Neubauer chamber and adjusted on a 96-well microplate to the concentration of 2 × 10^5^ cells/mL. The cells were cultivated in a supplemented RPMI 1640 medium at 37 °C with 5% CO_2_ atmosphere for 24 h. Next, CZ-EO that had been previously dissolved in DMSO was added to the wells at concentrations ranging from 6.25 to 400 µg/mL, and the cells were cultivated in the same previously described conditions for 48 h. Afterward, the cells were washed, and 20 µL of a PBS (phosphate-buffered saline) solution containing 7 mg of 3-(4,5-dimethylthiazol-2-yl)-2,5-diphenyltetrazolium bromide) (MTT) (Sigma-Aldrich, St. Louis, MO, USA) was added to each well. Cell viability was determined by the colorimetric metabolic activity assay, which assesses the ability that metabolically active cells have to reduce MTT by converting their yellow salts into purple formazan crystals. The plates were incubated at 37 °C and in a 5% CO_2_ atmosphere for 4 h, and the formazan precipitate was solubilized with 100 μL of isopropyl alcohol (Sigma-Aldrich, St. Louis, MO, USA). The absorbance was read at 550 nm with a spectrophotometer (Biochrom Corp., Miami, USA). The negative, solvent, and positive control groups consisted of LLCMK_2_ cells incubated with a RPMI 1640 medium containing 0.1% DMSO, and 10% DMSO, respectively. Experiments were performed in triplicate.

### 4.8. Statistical Analysis

All the experiments were performed in triplicate and repeated at least two times. The IC_50_ (the inhibitory concentration that inhibited trypomastigote and promastigote viability or intracellular amastigote growth by 50%) and CC_50_ (the CZ-EO that was cytotoxic to 50% of the cells) values were calculated by a non-linear regression dose-response inhibition curve. The selective index (SI), which indicates the parasite toxicity as compared to the host, was calculated as the ratio between CC_50_ and IC_50_ [49]. Data were analyzed by repeated measures of two-way analysis of variance followed by Dunnet’s comparison. Statistical analyses were performed using GraphPad Prism 5 (GraphdPad Software, San Diego, CA, USA).

## 5. Conclusions

The essential oil from *C. zizanioides* roots (CZ-EO) is effective against *Trypanosoma cruzi*, *Leishmania amazonensis*, and periodontopathogenic bacteria in vitro. Moreover, it displays moderate cytotoxicity to LLCMK_2_ cells and a very interesting selective index (SI) with regard to the *L. amazonensis* promastigote and amastigote forms and the *T. cruzi* trypomastigote form. These results suggest that CZ-EO can be potentially used as a component of new oral care products, and can be further exploited for the development of new antileishmanial and antitrypanosomal drugs. To this end, further in vitro studies aiming to elucidate the mode of action of CZ-EO and to identify the compounds underlying its antileishmanial and antitrypanosomal activities are needed, and the possible synergistic, additive or even antagonistic effects among them must be investigated.

## Figures and Tables

**Table 1:** CZ-EO chemical composition, as determined by GC-FID and GC-MS.

Compounds	RI^a^		%RA^b^
	Experimental	Literature [21]	
Naphthalene	1175	1179	0.6 ± 0.2
β-cubebene	1392	1390	1.8 ± 0.2
Isolongifolene	1406	1402	2.4 ± 0.2
β-Gurjunene	1434	1432	1.2 ± 0.1
α-Patchoulene	1452	1456	3.0 ± 0.2
β-Cadinene	1470	1473	3.6 ± 0.1
α-Muurolene	1475	1480	6.0 ± 0.1
α-Amorphene	1486	1485	1.2 ± 0.2
Valencene	1493	1491	0.6 ± 0.2
α-Bulnesene	1501	1505	4.4 ± 0.2
α-Elemol	1546	1547	0.6 ± 0.2
β-Vatirenene	1548	1554	0.6 ± 0.1
β-Eudesmol	1552	1548	10.8 ± 0.3
Spathulenol	1576	1576	4.0 ± 0.2
Guaiol	1598	1595	3.2 ± 0.1
Humulane-1,6-dien-3-ol	1618	1619	3.0 ± 0.3
Cubenol	1640	1642	3.4 ± 0.1
Agarospirol	1644	1639	3.0 ± 0.3
Cedren-13-ol	1657	1657	3.6 ± 0.1
Patchouli alcohol	1663	1659	5.6 ± 0.2
Khusimol	1747	1747	30.0 ± 0.3
Nootkatone	1802	1800	2.8 ± 0.1
Sesquiterpene hydrocarbons			24.6 ± 0.5
Oxygenated sesquiterpenes			70.4 ± 0.1
Not identified			5.6 ± 0.0

^a^**RI**: Retention Index; ^b^**RA**: relative area.

**Table 2:** Minimum Inhibitory Concentration (MIC) and Minimum Bactericidal Concentration (MBC) values (µg/mL) of CZ-EO and the positive control (Chlorhexidine) against periodontopathogenic bacteria.

Bacteria	CZ-EO	Chlorhexidine *
*P. gingivalis* ATCC 33277	62.5 ± 25.0/150.0 ± 58.0	7.4 ± 0.0/7.4 ± 0.0
*P. gingivalis* Clinical Isolate	100.0 ± 0.0/250.0 ± 100.0	7.4 ± 0.0/14.8 ± 0.0
*P. intermedia* ATCC 49046	22.0 ± 6.25/400.0 ± 0.0	14.8 ± 0.0/14.8 ± 0.0
*P. intermedia* Clinical Isolate	150.0 ± 58.0/400.0 ± 0.0	7.4 ± 0.0/14.8 ± 0.0
*P. nigrescens* ATCC 33563	62.5.0 ± 25.0/62.5 ± 25.0	7.4 ± 0.0/14.8 ± 0.0
*F. nucleatum* Clinical Isolate	250.0 ± 100.0/400.0 ± 0.0	0.9 ± 0.0/14.8 ± 0.0
*F. nucleatum* ATCC 25586	50.0 ± 0.0/50.0.0 ± 0.0	3.7 ± 0.0/3.7 ± 0.0
*P. melaninogenica* ATCC 700524	50.0 ± 0.0/50.0 ± 0.0	3.7 ± 0.0/29.5 ± 0.0
*A. actinomycetemcomitans* ATCC 43717	22.0 ± 6.25/22.0 ± 6.25	7.4 ± 0.0/7.4 ± 0.0

* Positive control.

**Table 3:** In vitro antileishmanial activity of CZ-EO against the L. amazonensis promastigote and amastigote forms.

	% of Lysis ± S.D/Concentration (μg/mL)					IC_50_ (μg/mL)
	50	25	12.5	6.25	3.12	
CZ-EO (promastigote form)	100 ± 0.00	100 ± 0.00	65.87 ± 5.90	57.12 ± 6.69	45.82 ± 3.81	7.20 ± 1.14
CZ-EO (amastigote form)	97.90 ± 0.16	68.41 ± 1.62	47.38 ± 2.00	36.50 ± 0.90	30.55 ± 0.80	16.21 ± 1.02
	**0.19**	**0.095**	**0.047**	**0.023**	**0.011**	
Amphotericin B *	44.38 ± 0.53	36.89 ± 0.79	33.61 ± 0.62	29.02 ± 1.85	23.50 ± 1.58	0.25 ± 0.39

* Positive control.

**Table 4:** In vitro trypanocidal of CZ-EO against the *Trypanosoma cruzi* trypomastigote form and its cytotoxicity to LLCMK_2_ cells.

	% of Lysis ± S.D/Concentration (μg/mL)										IC_50_ (μg/mL)
	200		100		50		25		12.5		
CZ-EO (trypomastigote form)	80.2 ± 6.1		91.6 ± 4.5		87.4 ± 1.2		92.0 ± 0.3		100.6 ± 0.5		11.2 ± 0.5
Benznidazole *	97.8 ± 1.0		95.2 ± 0.6		73.9 ± 4.3		75.0 ± 4.7		55.4 ± 1.0		9.9 ± 1.2
	**6.25**	**12.5**		**25**	**50**	**100**		**200**		**400**	**CC_50_** **(** **μg/mL)**
CZ-EO (LLCMK2 cells)	100 ± 0	100 ± 0		100 ± 0	100 ± 0	91.2 ± 6.0		86.7 ± 1.5		65.3 ± 2.5	565.4 ± 1.0

* Positive control.

## Data Availability

Data are contained within the article.
